# Association of Glycolysis-Enhancing α-1 Blockers With Risk of Developing Parkinson Disease

**DOI:** 10.1001/jamaneurol.2020.5157

**Published:** 2021-02-01

**Authors:** Jacob E. Simmering, Michael J. Welsh, Lei Liu, Nandakumar S. Narayanan, Anton Pottegård

**Affiliations:** 1 Department of Internal Medicine, Roy J. and Lucille A. Carver College of Medicine, University of Iowa, Iowa City; 2 Department of Neurology, Roy J. and Lucille A. Carver College of Medicine, University of Iowa, Iowa City; 3 Department of Molecular Physiology and Biophysics, Roy J. and Lucille A. Carver College of Medicine, University of Iowa, Iowa City; 4 Pappajohn Biomedical Institute, Roy J. and Lucille A. Carver College of Medicine, University of Iowa, Iowa City; 5 Howard Hughes Medical Institute, University of Iowa, Iowa City; 6 Center of Stroke, Beijing Institute for Brain Disorders, Advanced Innovation Center for Human Brain Protection, Capital Medical University, Beijing, China; 7 Department of Public Health, University of Southern Denmark, Odense, Denmark

## Abstract

**Importance:**

Parkinson disease (PD) is a common neurodegenerative disease. A treatment that prevents or delays development of PD is a critical unmet need. Terazosin and closely related drugs were recently discovered to enhance glycolysis and reduce PD progression in animal models and human clinical databases.

**Objective:**

To determine whether use of terazosin, doxazosin, and alfuzosin is associated with a decreased risk of developing PD.

**Design, Setting, and Participants:**

This cohort study used active comparator control and propensity score–matched data from Danish nationwide health registries, including the Danish National Prescription Registry, the Danish National Patient Registry, and the Danish Civil Registration System, from January 1996 to December 2017 and data from the Truven Health Analytics MarketScan database from January 2001 to December 2017. Men without PD who newly initiated terazosin/doxazosin/alfuzosin therapy or tamsulosin therapy, which is used for a similar indication (benign prostatic hyperplasia or unspecified urinary problems) but does not enhance glycolysis, and had at least 1 year of follow-up after medication start were included. In Denmark, the database included all residents, while the Truven database is a compilation of insurance claims across the US. Data were analyzed from February 2019 to July 2020.

**Exposures:**

Patients who used terazosin/doxazosin/alfuzosin vs tamsulosin. Additional dose-response analyses were carried out.

**Main Outcomes and Measures:**

Differences in the hazard of developing PD identified by diagnoses or use of PD-specific medications between patients who ever used terazosin/doxazosin/alfuzosin or tamsulosin.

**Results:**

A cohort of 52 365 propensity score–matched pairs of terazosin/doxazosin/alfuzosin and tamsulosin users were identified in the Danish registries, of which all were male and the mean (SD) age was 67.9 (10.4) years, and 94 883 propensity score–matched pairs were identified in the Truven database, of which all were male and the mean (SD) age was 63.8 (11.1) years. Patients in the Danish cohort who used terazosin/doxazosin/alfuzosin had a hazard ratio (HR) for developing PD of 0.88 (95% CI, 0.81-0.98), and patients in the Truven cohort had an HR of 0.63 (95% CI, 0.58-0.69). There was a dose-response association with short-duration, medium-duration, and long-duration use of terazosin/doxazosin/alfuzosin users having a decreasing HR in both the Danish cohort (short: HR, 0.95; 95% CI, 0.84-1.07; medium: HR, 0.88; 95% CI, 0.77-1.01; long: HR, 0.79; 95% CI, 0.66-0.95) and Truven cohort (short: HR, 0.70; 95% CI, 0.64-0.76; medium: HR, 0.58; 95% CI, 0.52-0.64; long: HR, 0.46; 95% CI, 0.36-0.57).

**Conclusions and Relevance:**

These data suggest that users of terazosin/doxazosin/alfuzosin are at lower hazard of developing PD compared with users of tamsulosin. Future work is needed to further assess this association.

## Key Points

**Question:**

Is use of glycolysis-enhancing drugs, such as terazosin, doxazosin, and alfuzosin, associated with decreased risk of Parkinson disease compared with use of tamsulosin, a drug prescribed for similar indications but which does not enhance glycolysis?

**Findings:**

In this cohort study of 147 248 propensity score–matched pairs of terazosin/doxazosin/alfuzosin users and tamsulosin users from Danish nationwide health registries and the Truven Health Analytics MarketScan, the use of terazosin/doxazosin/alfuzosin was associated with a 12% to 37% decrease in Parkinson disease risk compared with use of tamsulosin.

**Meaning:**

Use of terazosin/doxazosin/alfuzosin may lower the risk of developing Parkinson disease.

## Teaser

This cohort study investigates whether use of terazosin, doxazosin, and alfuzosin is associated with a decreased risk of developing Parkinson disease compared with use of tamsulosin.

## Introduction

Parkinson disease (PD) is a devastating neurodegenerative disease without any neuroprotective treatments.^1^ Currently, roughly 10 million individuals worldwide have PD, and the number is growing as the population ages. PD exhibits diverse manifestations, including motor and cognitive symptoms.^2,3^ Mainstay therapies, such as levodopa and brain stimulation, can improve motor symptoms of PD but do not slow progressive neurodegeneration.^4,5,6^ Despite intensive research, however, potential interventions have not demonstrated consistent neuroprotection.^7,8,9^ Thus, people with PD contend with inexorable and irreversible neurodegeneration and the resulting morbidity and mortality.

Impaired energy metabolism is a potential and largely unexplored pathogenic factor in PD.^10^ Age is the major risk factor for PD, and increased age is associated with impaired cerebral glucose metabolism, reduced mitochondrial biogenesis, and lower adenosine triphosphate (ATP) levels.^11,12^ Among people with PD, glycolysis and mitochondria function are decreased.^13,14,15,16,17^ Moreover, mitochondrial toxins, such as MPTP (1-methyl-4-phenyl-1,2,3,6-tetrahydropyridine) and rotenone, induce PD and PD-like phenotypes.^18^ In addition, variants that cause familial PD often disrupt mitochondrial function and energy balance (eg, *PINK1*, *parkin*, *DJ-1*, *CHCHD2*, *α-synuclein*, *LRRK2*).^19,20^ These findings suggest that impaired energy metabolism plays a role in the pathogenesis of PD. By extension, therapies that increase energy metabolism and ATP might prevent the development of PD.

An opportunity to test this idea came when biochemical, functional, and structural studies showed that terazosin (TZ) binds and increases the activity of phosphoglycerate kinase 1 (PGK1), the first ATP-generating enzyme in glycolysis.^21^ TZ crosses the blood-brain barrier, increasing glycolysis and ATP in brain cells.^22^ Studies in toxin-induced and genetic models of PD in mice, rats, flies, and cells revealed that TZ slowed or prevented neuron loss.^22^

TZ is an α1-adrenergic receptor antagonist that is commonly used to manage benign prostatic hyperplasia and, rarely, hypertension.^23^ The effects of TZ on PGK1 are independent of effects on α1-adrenergic receptors.^21^ Two other closely related α1-adrenergic antagonists, doxazosin (DZ) and alfuzosin (AZ), have a similar PGK1 binding motif, enhance energy metabolism, and are efficacious in a PD model.^22^ Tamsulosin is another α1-adrenergic receptor antagonist. However, tamsulosin is structurally unrelated to TZ, does not enhance energy metabolism, and lacks effect in a PD model.^22^

Tamsulosin is also commonly prescribed for the same indication as TZ/DZ/AZ, benign prostatic hyperplasia, and crosses the blood-brain barrier^24^ but lacks the interaction with PGK1 that enhances glycolysis,^22^ providing a unique opportunity to use tamsulosin as a control in testing whether TZ/DZ/AZ benefits people with PD. This provides powerful protection against confounding by indication, as the users of TZ/DZ/AZ and tamsulosin should be very similar when treatment is initiated. This should reduce the risk of finding a difference between the 2 cohorts due to one group having a lower risk of PD because of some unobserved confounder and also selecting into the treatment group by the same factor. We previously found that relative to those who used tamsulosin or no α1-adrenergic antagonist, people with PD who used TZ/DZ/AZ had slower progression of motor dysfunction and reduced PD-related complications.^22^ For the subset of patients with PD for whom impaired energy metabolism played a pathogenic role, these results suggested that the neuroprotective effect of enhancing PGK1 activity and glycolysis might prevent development or delay PD. Specifically, we hypothesized that patients newly beginning TZ/DZ/AZ therapy would have a lower hazard of developing PD compared with patients newly beginning tamsulosin therapy. We tested this hypothesis using data from 2 large administrative health care databases from the US and Denmark.

## Methods

We performed 2 conceptually similar investigations in the US and Denmark. However, owing to differences in the data sources between the 2 countries, differences in PD treatment and recording, and social and health system differences, the designs are not strictly parallel. All analysis was performed on fully deidentified secondary databases and was therefore exempt from institutional review board approval. This article followed the Strengthening the Reporting of Observational Studies in Epidemiology (STROBE) reporting guideline.

### Databases

We used health care utilization data from 2 sets of independent databases: (1) Danish nationwide health registries, including the Danish National Prescription Registry,^25^ the Danish National Patient Registry,^26^ and the Danish Civil Registration System,^27^ from January 1996 to December 2017 and (2) the Truven Health Analytics MarketScan database from January 2001 to December 2017. We included individuals from the time of medication start until they exited the database or December 2017, whichever occurred first. These databases are described in detail in eMethods 1 in the Supplement.

### Exposure Definitions

We identified exposure to the medications of interest using the anatomical therapeutic chemical (ATC) codes in the Danish cohort and National Drug Code (NDC) numbers in the Truven cohort. Full ATC and NDC codes are listed in eMethods 2 in the Supplement. Specifically, we defined enrollees with claims or prescription fill events for TZ, DZ, or AZ as users of TZ/DZ/AZ and enrollees with claims or prescription fill events for tamsulosin as users of tamsulosin. We excluded any enrollees who met our outcome definitions before or within 1 year of starting the medication. As use of TZ/DZ/AZ and tamsulosin are rare in women, we only included men. Further, as both PD and prostate hyperplasia are uncommon in young individuals, we restricted our analysis to those 40 years or older at medication start.

We observed each individual starting 1 year after the initiation of TZ/DZ/AZ or tamsulosin, as it was considered unlikely that any effect on PD risk would be immediately evident. We further required that all patients have at least 2 prescription fills within the first year of use (that is, before the start of follow-up) to be included in the study, reasoning that patients with only 1 prescription fill likely discontinued therapy and their exposure duration was not well measured by the number of prescription fills and number of doses supplied. We censored anyone who switched between drugs in the TZ/DZ/AZ and tamsulosin groups at the time of the switch to isolate the effect of the drugs of interest. Finally, in the Danish registry, anyone with a history of having used other α-blocking medications at baseline were excluded. The full set of ATC codes are listed in eMethods 2 in the Supplement.

To ensure identified users were new users of the medication, we required a lookback period. A limitation of administrative data is that medication dispensing events during the early period of enrollment may either be a new prescription or a refill of an existing treatment, and the claims data do not distinguish between the 2 event types. To address this issue, prior to the first prescription date, we required at least 12 continuous months of enrollment with prescription drug coverage in the Truven cohort and excluded anyone who immigrated in the prior 24 months in the Danish cohort.

We described the cumulative amount of therapy using defined daily doses (DDD). DDD is a metric that measures both the duration and strength of therapy by normalizing the dose by the medication-specific defined average dose as determined by the World Health Organization.^28^ In our analysis, we binned the cumulative amount of DDDs filled into categories comprising low use of 1 to 499 DDDs, moderate use of 500 to 1999 DDDs, and long-term use of 2000 or more DDDs. We included all prescription fills during the observation period.

### Outcome Definitions

Our primary outcome was the development of PD during the observation window. In the Danish database, the event was defined as a hospital diagnosis of PD (*International Statistical Classification of Diseases and Related Health Problems, Tenth Revision *[*ICD*-*10*] code G20) or any dispensing event for the anti-PD medications levodopa (ATC code N04BA01), orphenadrine (ATC code N04AB02), or selegiline, rasagiline, or safinamide (ATC codes N04BD01, N04BD02, and N04BD03). In the Truven database, the event was defined as the first diagnosis of PD (*ICD*-*9* code 332.0 or *ICD*-*10* code G20) in any setting (inpatient, emergency department, outpatient) or a dispensing event for levodopa (NDC codes listed in eMethods 2 in the Supplement). The set of medications used differs between the analysis of the Danish and Truven cohorts due to differences in PD management between the 2 countries. The case definitions were based on previously used and validated *ICD*-*9* diagnosis codes and medications in the US^29,30^ and was modified by a Danish neurologist to better reflect Danish clinical practice. In people who met our outcome definition, we defined the event date as the first date with a relevant diagnosis or medication dispensing event.

### Matching

Propensity score matching was conducted to ensure similarity between the TZ/DZ/AZ and tamsulosin groups on other potential risk factors. The Danish cohort was matched on a propensity score based on age at medication start; year of medication start; use of any low-dose aspirin, nonsteroidal anti-inflammatory drugs, 5-α reductase inhibitors, statins, or selective serotonin reuptake inhibitors in the prior 120 days; diagnosis of diabetes, chronic obstructive pulmonary disease, heart failure, or alcohol-related disease in the prior 5 years; Charlson Comorbidity Index score (0, low; 1 to 2, medium; 3 or more, high) based on the prior 5 years; and highest education level (10 or fewer years, 11 to 12 years, and 13 or more years).

A similar process was done in the Truven database. We developed a propensity score incorporating age at medication start, each of the Agency for Healthcare Research and Quality/Elixhauser comorbidities^31^ observed during the premedication period, the year of medication start, the number of hospitalizations, number of hospital days, and number of outpatient visits during the prior year. In the event of a tie with more than 1 tamsulosin user being an equally close match to the TZ/DZ/AZ user, we selected the tamsulosin match at random. Each tamsulosin user was matched only once.

For both cohorts, the top and bottom 2.5% were trimmed^32^ and the remaining patients were matched 1:1 for TZ/DZ/AZ and tamsulosin use. As the follow-up period in the Truven cohort was variable, we conditioned matches on having a similar follow-up duration (within 30 days) between the TZ/DZ/AZ user and the tamsulosin match. No matching on follow-up was done in the Danish registry–derived cohort, as follow-up periods were generally much longer and less variable. In both cohorts, the quality of the matches was assessed by comparing the absolute and standardized mean difference between TZ/DZ/AZ and tamsulosin users.

### Statistical Analysis

The primary analysis in both cohorts conformed to an intention-to-treat analysis, comparing ever use (2 or more prescriptions) of TZ/DZ/AZ to ever use (2 or more prescriptions) of tamsulosin. The fraction remaining free of PD over time was estimated using Kaplan-Meier curves. We quantified the survival difference using Cox proportional hazards regression to estimate the hazard ratio (HR) for PD among users of TZ/DZ/AZ compared with users of tamsulosin. Additionally, as a sensitivity analysis for dose response, we allowed a time-varying effect of TZ/DZ/AZ. Specifically, we used Cox proportional hazards models after stratifying all follow-up by the cumulative number of DDDs to arrive at estimates of the effect of TZ/DZ/AZ by the duration of therapy. In these analyses, patients taking tamsulosin remained the reference group. Finally, a post hoc analysis was performed interacting medication group and age at medication initiation (70 years and older vs younger than 70 years) to assess the potential for a greater protective effect in older individuals.

Cohort demographic characteristics were compared using *t* tests (continuous variables) or χ^2^ tests (categorical variables). HRs were tested using the Wald statistic from the Cox proportional hazards regression. All hypothesis tests were 2-sided with the level of significance defined at *P* < .05. Analysis of the Danish cohort was performed using Stata MP version 16.1 (StataCorp), while analysis of the Truven cohort was performed using R version 3.5.1 (The R Foundation).

## Results

A cohort of 52 365 propensity score–matched pairs of TZ/DZ/AZ and tamsulosin users were identified in the Danish registries, of which all were male and the mean (SD) age was 67.9 (10.4) years, and 94 883 propensity score–matched pairs were identified in the Truven database, of which all were male and the mean (SD) age was 63.8 (11.1) years. Full cohort size by exclusion step is reported in eTable 1 in the Supplement. After propensity score matching, the standardized mean differences indicated balancing on the measured covariates in both cohorts, suggesting suitability for direct comparison in the survival analysis. Baseline covariates, both before and after matching, were well balanced in the Danish and Truven cohorts (eTables 2 and 3 in the Supplement). Summary of selected demographic characteristics, number of DDDs, and outcomes for both cohorts are reported in the Table. A breakdown of the sample size and cumulative number of DDDs for each medication is reported in eTable 4 in the Supplement.

**Table.** Selected Demographic, Exposure, and Follow-up Measures

Measure	Median (IQR)			
	Danish registries^a^		Truven Health Analytics MarketScan	
	TZ/DZ/AZ users	Tamsulosin users	TZ/DZ/AZ users	Tamsulosin users
Sample size, No.	52 365	52 365	94 883	94 883
Age at medication start, y	68 (61-75)	68 (61-75)	62 (56-72)	62 (56-72)
Time of follow-up, y	4.99 (2.29-8.82)	5.35 (2.49-9.34)	2.91 (1.77-5.01)	2.91 (1.78-5.01)
Cumulative standardized dose^b^	780 (240-1988)	780 (230-1920)	406 (139-976)	493 (176-1108)

Abbreviations: AZ, alfuzosin; DZ, doxazosin; IQR, interquartile range; TZ, terazosin.

^a^ Danish nationwide health registries included the Danish National Prescription Registry, the Danish National Patient Registry, and the Danish Civil Registration System.

^b^ Defined as 1 equals the average daily dose for each drug.

In the Danish cohort, 798 TZ/DZ/AZ users developed PD (2.6 per 1000 person-years) compared with 939 tamsulosin users (2.9 per 1000 person-years). In the Truven cohort, a total of 862 TZ/DZ/AZ users developed PD (2.40 per 1000 person-years), and 1368 tamsulosin users developed PD (3.81 per 1000 person-years). While the designs and outcome definitions used in the analyses were roughly parallel, differences between the 2 countries’ health care systems and coding practices make direct comparison between the Truven cohort and the Danish cohort difficult.

**Figure 1.**
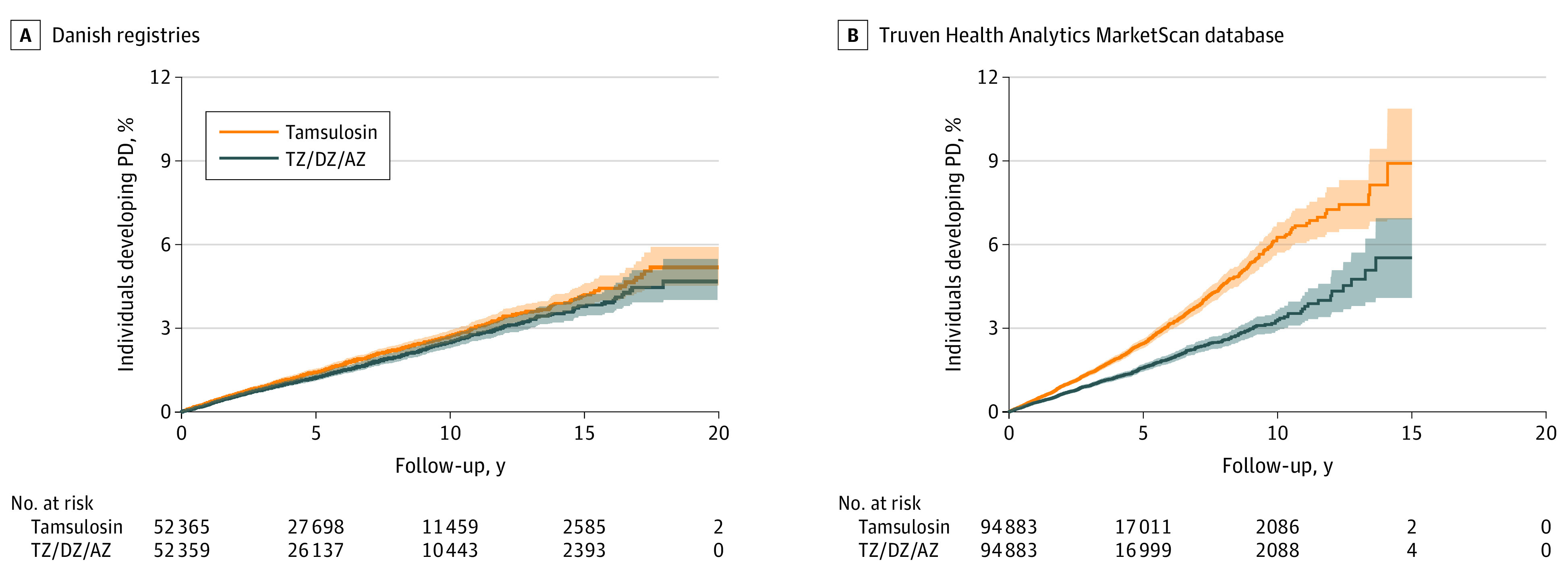
Cumulative Incidence of Parkinson Disease (PD) in the Danish and Truven Cohorts A, Kaplan-Meier plot of Danish nationwide health registries, including the Danish National Prescription Registry, the Danish National Patient Registry, and the Danish Civil Registration System. B, Kaplan-Meier plot of Truven Health Analytics MarketScan database. The lines denote the Kaplan-Meier estimated cumulative incidence of PD by cohort while the shaded regions denote the 95% CIs. The Truven cohort terminates at 15 years, as that is the longest possible follow-up in that database, while the Danish cohort has 5 additional years of follow-up possible. In both samples, use of terazosin (TZ), doxazosin (DZ), or alfuzosin (AZ) was associated with lower cumulative incidence of PD compared with use of tamsulosin.

Kaplan-Meier cumulative incidence curves are shown in Figure 1. Cox proportional hazard regression found evidence of a difference in hazard. Among the Danish residents, those taking TZ/DZ/AZ had a 12% lower hazard of developing PD compared with those taking tamsulosin (HR, 0.88; 95% CI, 0.81-0.98). A directionally similar and larger association was observed in the Truven database, with enrollees taking TZ/DZ/AZ having a 37% reduction in hazard compared with those taking tamsulosin (HR, 0.63; 95% CI, 0.58-0.69).

We assessed our Cox model for violation of the proportional hazards assumption. The Kaplan-Meier curves in Figure 1 and the log(−log[survival]) curves in eFigure 2 in the Supplement suggest that the proportional hazards assumption is reasonable in both data sets. There was no significant correlation between the Schoenfeld residuals and time in the Danish cohort. There was a small correlation that was statistically significant in the Truven cohort (*r* = −0.06; *P* = .006); however, this is likely the result of the large sample creating very high power to detect deviations from the proportional hazard assumption and unlikely to influence the estimated HR.

**Figure 2.**
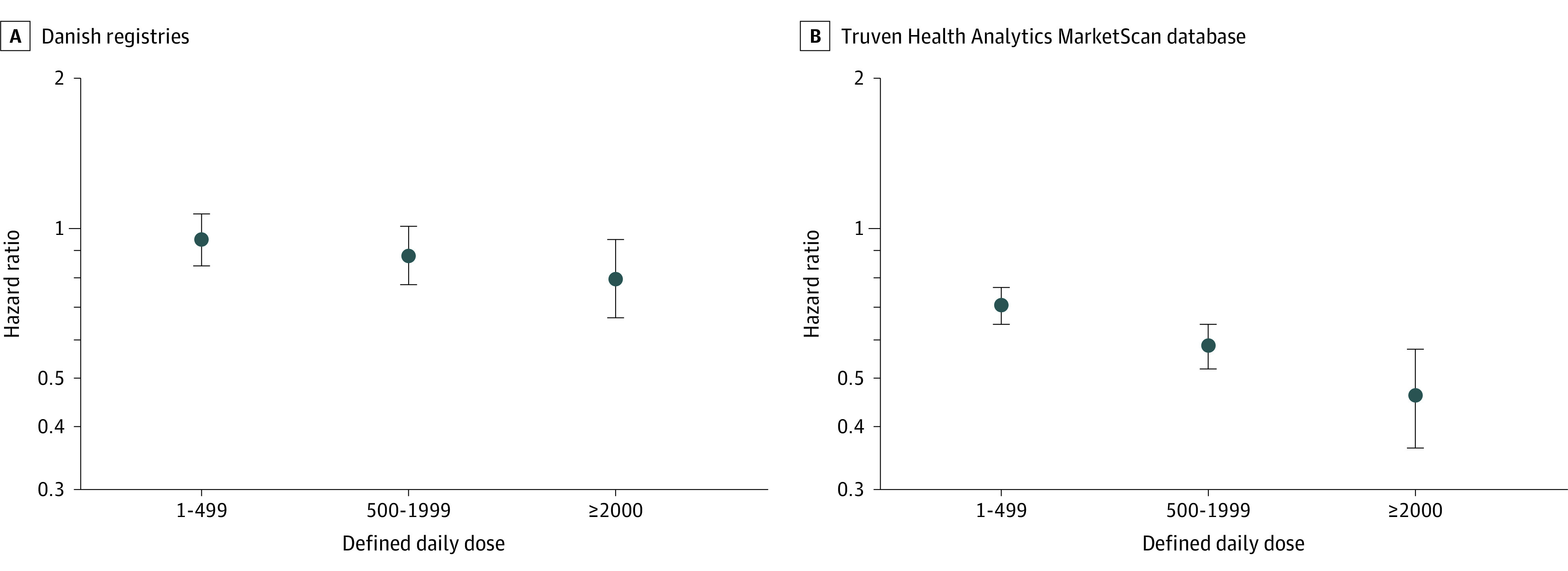
Estimated Hazard Ratio by Cumulative Number of Defined Daily Doses A, Estimated hazard of developing Parkinson disease among those in the Danish nationwide health registries, including the Danish National Prescription Registry, the Danish National Patient Registry, and the Danish Civil Registration System. B, Estimated hazard of developing Parkinson disease among those in the Truven Health Analytics MarketScan database. Values are hazard ratios, and error bars indicate 95% CIs. Increasing intensity and duration of treatment as measured by defined daily doses was associated with a decreasing estimated hazard ratio.

The observed difference among those who had ever used TZ/DZ/AZ vs tamsulosin varied with time taking treatment (Figure 2). In both cohorts, the estimated HR for developing PD in those taking TZ/DZ/AZ compared with tamsulosin reduced with increased cumulative prescriptions filled. Both cohorts had directionally similar HRs following a dose-response pattern for the short-term (Danish cohort: HR, 0.95; 95% CI, 0.84-1.07; Truven cohort: HR, 0.70; 95% CI, 0.64-0.76), medium-term (Danish cohort: HR, 0.88; 95% CI, 0.77-1.01; Truven cohort: HR, 0.58; 95% CI, 0.52-0.64), and long-term (Danish cohort: HR, 0.79; 95% CI, 0.66-0.95; Truven cohort: HR, 0.46; 95% CI, 0.36-0.57) cumulative use of TZ/DZ/AZ compared with tamsulosin.

In the Danish database, the reduction in HR was seemingly larger among people 70 years and older (HR, 0.83; 95% CI, 0.73-0.94) than for those younger than 70 years (HR, 0.95; 95% CI, 0.83-1.09), but this difference did not reach statistical significance (*P* = .18). A similar pattern was observed in the Truven cohort, with the greatest reduction in HR being among those 70 years or older (HR, 0.63; 95% CI, 0.58-0.68) compared with those younger than 70 years (HR, 0.68; 95% CI, 0.61-0.76), but again, the difference between the groups was not significant (*P* = .38). Kaplan-Meier curves for both cohorts are shown in eFigure 1 in the Supplement.

## Discussion

These results indicate that men using TZ/DZ/AZ had hazards of developing PD that were 12% to 37% lower than men taking tamsulosin. Longer use of TZ/DZ/AZ was associated with a greater reduction in the observed hazard relative to those using tamsulosin for a similar duration. These findings add to our prior study focused on TZ/DZ/AZ and PD, reporting that TZ/DZ/AZ slowed or prevented neurodegeneration in models of PD and that use of TZ/DZ/AZ was associated with slower progression and fewer complications in people with PD.^22^

Potential molecular mechanisms for these effects of TZ/DZ/AZ are identified by earlier work. TZ/DZ/AZ enhances PGK1 activity, thereby increasing glycolysis and cellular ATP levels.^21,22^ These changes might prevent PD neurodegeneration in at least 2 ways. First, PD exhibits deficits in energy metabolism, as evidenced by the aging-related decline in ATP and energy metabolism, the genetic defects in mitochondrial function, and the toxin inhibition of mitochondrial function.^10,33^ By enhancing glycolysis and increasing ATP levels, TZ/DZ/AZ may counteract these deficits. Second, it is hypothesized that aggregates of α-synuclein induce neurodegeneration in PD.^34,35^ TZ/DZ/AZ elevates ATP, a hydrotrope that may prevent aggregate formation and dissolve previously formed aggregates.^36,37^ Therefore, if the hypothesized α-synuclein and neurodegeneration relationship is correct, TZ/DZ/AZ may reduce PD risk by preventing aggregate-induced neurodegeneration. However, assessment of this hypothesized mechanism is beyond the scope of this study. A combination of these and other mechanisms are also possible, including ATP-dependent disaggregates and chaperones that reduce apoptosis.

This study has advantages. Principally, we analyzed 2 large, independent, and international databases. Differences between the databases add strength to our analyses. The Truven database has the advantage of a larger and more diverse cohort than the Danish cohort. The Danish cohort has the advantage of complete capture of the population, and this population’s access to health care is significantly more uniform than in the US. The consistency of the results between these databases, especially given the cultural and health system differences between the US and Denmark, suggest that the effect is reproducible across populations.

### Limitations

These results, while suggestive, are not without limitations, chiefly due to the observational nature of our study. The Truven database is derived from private insurance plans, resulting in loss to follow-up when people change health insurance, greater variability and access across plans, and systemic differences between insured and uninsured populations. While these weaknesses are not shared with the Danish registries, this data source has its own limitations. In particular, the Danish registries lack information about nonhospital diagnoses. These cross-database and cross-national differences prevent a strict replication and force some unavoidable differences in design. Both sources have the issues common to administrative data. Our outcome depends on the accurate and timely diagnosis of PD. However, there is no a priori reason to suspect that PD diagnosis or therapy would be delayed in one treatment group and not the other, and as such, these concerns are somewhat mitigated by the use of the active comparator design. Additionally, the use of medication dispensing events for anti-PD medications is a proxy event—it is possible the medication was being used for a non-PD indication. While we used propensity score matching to pair users of TZ/DZ/AZ and tamsulosin, it remains possible that unobserved differences between the groups exist. An additional limitation is a lack of data on several risk factors for PD, like head trauma or pesticide exposure, that are not captured by our data resources. However, this would only confound our estimates if the rates of unobserved exposure differ between the TZ/DZ/AZ and tamsulosin groups, which we consider unlikely.

## Conclusions

The combination of the strength of these results from 2 large databases in 2 different countries with different cultural and health care systems, the prior mechanistic studies, the results in animal models of PD, and the prior observational results from patients with PD provide compelling evidence that glycolysis-enhancing drugs might be neuroprotective and prevent or delay the development of PD. Given the heterogeneous pathophysiology of PD, it may be important to identify people with impaired energy metabolism or those who might benefit from glycolysis-enhancing drugs. As such, future investigations are needed to identify whether a specific subset of patients are more likely to benefit from treatment. Regardless of such considerations, future work, especially in the form of randomized trials, are needed to fully resolve questions related to the causal effect of TZ/DZ/AZ.

## Notes

Supplement. **eMethods 1.** Details about Danish nationwide registries. **eMethods 2.** Drug identification codes. **eTable 1.** Cohort sample size by exclusion step. **eTable 2.** Demographic and baseline characteristics of the Danish cohort before and after matching. **eTable 3.** Demographic and baseline characteristics of the Truven cohort before and after matching. **eTable 4.** Typical doses and duration of therapy by drug. **eFigure 1.** Kaplan-Meier survival curves stratified by age at medication start. **eFigure 2.** Log(−log[survival]) plots.
