# Supplementary material for: Association of Glycolysis-Enhancing α-1 Blockers With Risk of Developing Parkinson Disease
Source: JAMA Neurol. 2021 Feb 1;78(4):1–7. doi: 10.1001/jamaneurol.2020.5157 (PMC7851758; doi:10.1001/jamaneurol.2020.5157)
Supplement: Supplement. — eMethods 1. Details about Danish nationwide registries. eMethods 2. Drug identification codes. eTable 1. Cohort sample size by exclusion step. eTable 2. Demographic and baseline characteristics of the Danish cohort before and after matching. eTable 3. Demographic and baseline characteristics of the Truven cohort before and after matching. eTable 4. Typical doses and duration of therapy by drug. eFigure 1. Kaplan-Meier survival curves stratified by age at medication start. eFigure 2. Log(−log[survival]) plots. [file jamaneurol-e205157-s001.pdf]

Supplemental Online Content

Simmering JE, Welsh MJ, Liu L, Narayanan NS, Pottegård A. Association of glycolysis-enhancing α-1 blockers with risk of developing Parkinson disease. *JAMA Neurol*. Published online February 1, 2021. doi:10.1001/jamaneurol.2020.5157

eMethods 1. Details about Danish nationwide registries
eMethods 2. Drug identification codes
eTable 1. Cohort sample size by exclusion step
eTable 2. Demographic and baseline characteristics of the Danish cohort before and after matching
eTable 3. Demographic and baseline characteristics of the Truven cohort before and after matching
eTable 4. Typical doses and duration of therapy by drug
eFigure 1. Kaplan-Meier survival curves stratified by age at medication start
eFigure 2. Log(−log[survival]) plots

This supplementary material has been provided by the authors to give readers additional information about their work.

© 2021 Simmering JE et al. *JAMA Neurology*.

eMethods 1. Details about Danish nationwide registries

Healthcare utilization data was abstracted from 3 Danish nationwide registries: the National Prescription Registry, the National Patient Register, and the Civil Registration System. Virtually all medical care in Denmark is furnished by the national health authorities, allowing population-based register linkage studies covering all legal residents of Denmark. Data was used from 1997 to 2016.

The Danish National Prescription Registry contains data, including date, dose and days supplied, for all prescription drugs dispensed at community pharmacies (e.g., it does not include inpatient medication usage) for Danish citizens since 1995. The medications are categorized according to the Anatomic Therapeutic Chemical (ATC) index, a hierarchical classification system developed by the WHO.

The National Patient Register contains nationwide data on all non-psychiatric hospital admissions from 1997 as well as all psychiatric inpatient, both non- and psychiatric outpatient, and emergency room contacts since 1995. Diagnoses were encoded in ICD-10 from 1994 to present.

The Civil Registration System contains data on date of death and migration to and from Denmark, allowing us to track all of the subjects.

Data was linked between the databases using the personal identification number, a unique identifier assigned to all Danish residents since 1968.

© 2021 Simmering JE et al. *JAMA Neurology*.

eMethods 2. Drug identification codes

Danish Registry
The Danish cohort has medication information encoded using the ATC.

The study drugs were terazosin (G04CA03), doxazosin (C02VA04), alfuzosin (G04CA01, G04CA51), and tamsulosin (G04CA02 and G04CA52-53). Other alpha blockers were those under ATC codes C02C* and G04CA* that were not one of the study drugs.

Medications included in the propensity score matching were low-dose aspirin (B01AC06, B01AC30, N02BA01, N02BA51), NSAIDs (M01A except M01AX), 5-alpha reductase inhibitors (G04CB01, G04CB02), statins (C10AA, C10BA02), and SSRIs (N06AB).

Additionally, the comorbidity calculations included medication use as well as diagnoses codes. Specifically, diabetes mellitus (A10), COPD (R03BB) and alcohol related disease (N07BB).

Truven Cohort
Identification of medications in the Truven cohort was done by NDC number. Whereas the ATC codes used in the Danish registry include one code per drug, the NDC encodes the drug, dose, route, manufactory, and other information. As a result, the number of NDC numbers for each drug is much greater.

The NDC codes used in this study were identified by matching on the generic name in Thompson Reuters Red Book. They are:

Alfuzosin
00024420010, 00378500505, 00378500577, 13668002101, 13668002105, 13668002130, 13668002164, 16590027930, 24987020010, 24987020020, 29300015501, 29300015519, 31722030201, 31722030205, 33261099430, 35356028100, 42291011001, 42291011010,
47335095618, 47335095681, 47335095688, 54569557100, 54868504600, 54868504601, 54868504602, 54868632900, 57237011490, 59212020010, 60429034701, 60505285001, 61919034930, 64679073802, 64679073803, 65862024901, 68071080530, 69097084407, 69097084412, 76282030201, 76282030205

Doxazosin
00049271030, 00049272030, 00049275041, 00049275066, 00049276041, 00049276066, 00049277041, 00049277066, 00049278041, 00049278066, 00093206701, 00093206801, 00093206901, 00093207001, 00093812001, 00093812019, 00093812093, 00093812101, 00093812119, 00093812193, 00093812201, 00093812219, 00093812293, 00093812301, 00172368560, 00172368570, 00172368660, 00172368670, 00172368680, 00172368760, 00172368770, 00172368780, 00172368860, 00172368870, 00228264211, 00228264250, 00228264311, 00228264350, 00228264411, 00228264450, 00228264511, 00228264550, 00378402101, 00378402201, 00378402401, 00378402801, 00403059701, 00403059730, 00403534130, 00403535530, 00591064001, 00591064101, 00781500101, 00781500110, 00781500201, 00781500210, 00781500301, 00781500310, 00781500401, 00781500410, 00904552260, 00904552261, 00904552360, 00904552361, 00904552460, 00904552461, 00904552560, 00904614060, 00904614080, 00904614160, 00904614180, 00904614260, 00904614280, 00904614360, 00904614380, 10544029290, 10544062990, 12280021300, 16590027430, 16729021101, 16729021117, 16729021201, 16729021217, 16729021301, 16729021317, 16729021401, 16729021417, 16729041401, 16729041417, 16729041501, 16729041517, 23490547503, 23490547601, 33261065800, 33261065830, 33261065860, 33261065890, 33261065900, 33261065930, 33261065990, 33358011630, 35356092730, 35356092790, 35356097530, 35356097560, 35356097590, 42254007530, 42254021030, 42254027530, 42291025810, 42291025901, 42291026001, 43063073990, 49884055201, 49884055210, 49884055301, 49884055310, 49884055401, 49884055410, 49884055501, 49884055510, 49999020630, 49999020690, 50111065101, 50111065102, 50111065201, 50111065202, 50111065203, 50111065301, 50111065302, 50111065303, 50111065401, 50111065402, 50268027311, 50268027315, 50268027411, 50268027415, 50268027511, 50268027515, 50268027611, 50268027615, 51079095701, 51079095720, 51079095801, 51079095820, 51079095901, 51079095920, 51927489600, 52544063901, 52544063905, 52544064001, 52544064005, 52544064101, 52544064105,

© 2021 Simmering JE et al. *JAMA Neurology*.

52544064201, 52544064205, 54569325000, 54569329700, 54569329701, 54569486400, 54569486500, 54569516800, 54569516900, 54569516901, 54569517000, 54569517001, 54569524500, 54569524501, 54868176801, 54868176802, 54868215100, 54868215101, 54868215102, 54868264001, 54868341900, 54868341901, 54868476700, 54868476701, 54868476702, 54868480100, 54868480101, 54868480102, 54868480103, 54868480200, 54868480201, 54868480202, 54868480203, 54868480204, 54868489500, 54868489501, 55045305300, 55045313000, 55045313008, 55045316100, 55045316108, 55289002290, 55289058430, 55289060030, 55289060090, 55700018730, 55700018790, 55700018930, 55700018990, 55887050630, 55887050660, 55887050682, 55887050690, 55887050730, 55887050760, 55887050790, 57866631001, 57866631002, 57866631003, 57866631004, 57866631101, 57866631102, 57866631103, 57866631104, 57866631201, 57866631202, 57866631203, 57866631204, 57866661901, 57866661902, 57866661903, 57866661904, 58016013030, 58016062500, 58016062502, 58016062530, 58016062560, 58016062590, 58016064400, 58016064402, 58016064430, 58016064460, 58016064490, 58016099700, 58016099702, 58016099730, 58016099760, 58016099790, 58177026604, 58177026608, 58177026622, 58177026704, 58177026708, 58177026722, 58177026804, 58177026808, 58177026822, 58177026904, 58177026908, 58864080990, 58864085630, 58864086030, 59762231006, 59762232006, 59762234006, 59762238006, 60429005301, 60429005310, 60429005401, 60429005410, 60429005501, 60429005510, 60429005601, 60429005610, 60429095301, 60429095310, 60429095401, 60429095410, 60429095501, 60429095510, 60429095601, 60429095610, 60505009300, 60505009301, 60505009400, 60505009401, 60505009408, 60505009500, 60505009501, 60505009505, 60505009508, 60505009600, 60505009601, 60505009608, 60760000730, 60760000790, 63629130901, 63629135501, 63629135502, 63629135503, 63629135504, 63629403701, 63629403702, 63629403703, 66116018930, 66336068230, 67253038010, 67253038011, 67253038015, 67253038110, 67253038111, 67253038115, 67253038210, 67253038211, 67253038215, 67253038310, 67253038311, 67253038315, 67253038410, 67253038411, 67253038415, 67253038510, 67253038511, 67253038515, 67253038610, 67253038611, 67253038615, 67253038710, 67253038711, 67253038715, 68084083611, 68084083621, 68084083625, 68084083695, 68084085111, 68084085121, 68084085125, 68084085195, 68084086211, 68084086221, 68084086225, 68084086295, 68115012130, 68115012230, 68115012260, 68115012330, 68258600803, 68258609909, 68258699603, 68258699609, 68645017054, 68645046354, 68645046370, 68645046454, 68645046470, 68645046554, 68645046570

Terazosin
00074332211, 00074332213, 00074332253, 00074332311, 00074332313, 00074332353, 00074332411, 00074332413, 00074332453, 00074332511, 00074332513, 00074332553, 00074380511, 00074380513, 00074380611, 00074380613, 00074380711, 00074380713, 00074380811, 00074380813, 00093076001, 00093076101, 00093076201, 00093076301, 00093433601, 00093433605, 00093433701, 00093433710, 00093433801, 00093433810, 00093433901, 00093433905, 00172433610, 00172433660, 00172433670, 00172433710, 00172433760, 00172433770, 00172433780, 00172433810, 00172433860, 00172433870, 00172433880, 00172433910, 00172433960, 00172433970, 00339653312, 00339653412, 00339653512, 00339653612, 00378157001, 00378226001, 00378226401, 00378226801, 00403488101, 00403488130, 00615133853, 00615133863, 00781154101, 00781154110, 00781155101, 00781155110, 00781156101, 00781156110, 00781157101, 00781157110, 00781205101, 00781205105, 00781205201, 00781205205, 00781205301, 00781205305, 00781205401, 00781205405, 00904548260, 00904548360, 00904548460, 00904548560, 00904565060, 00904565061, 00904565160, 00904565161, 00904565260, 00904565261, 00904565360, 00904565361, 00904612561, 00904612661, 00904612761, 00904612861, 10544028930, 10544028990, 21695081230, 21695081290, 21695081330, 21695081390, 23490681403, 23490681503, 33261021100, 33261021103, 33261021130, 33261021190, 33261021200, 33261021230, 33261021260, 33261021290, 33261087000, 33261087030, 35356096230, 35356096260, 35356096290, 38779219203, 38779219204, 38779219206, 49999022700, 49999022730, 49999022760, 49999022790, 51079093601, 51079093620, 51079093701, 51079093720, 51079093801, 51079093820, 51079093920, 51655067552, 51655067652, 51927326800, 52959082805, 52959082860, 52959082960, 53217021230, 53217021290, 54569174101, 54569285900, 54569285901, 54569285902, 54569380500, 54569380501, 54569405100, 54569405101, 54569406200, 54569406201, 54569411100, 54569419600, 54569419601, 54569419602, 54569487300, 54569487301, 54569487400, 54569487500, 54569487501, 54569487600, 54569487601, 54569855200, 54569857600, 54868366200, 54868366202, 54868366203, 54868384200, 54868422800, 54868422801, 54868422802, 54868424700, 54868424701, 54868424702, 54868424800, 54868424801, 54868424802, 54868424900, 54868424901, 54868424902, 54868424903, 54868480300, 54868480301,

© 2021 Simmering JE et al. *JAMA Neurology*.

55045283408, 55045289708, 55045310400, 55045310408, 55048079130, 55048079230, 55048079330, 55048079430, 55289004230, 55289007030, 55289019430, 55289035330, 55887048130, 55887048160, 55887048190, 55887048230, 57866648601, 57866648701, 57866648702, 57866648801, 57866648802, 57866648901, 58016043100, 58016043102, 58016043130, 58016043160, 58016043190, 58016067300, 58016067330, 58016067360, 58016067390, 58016067400, 58016067402, 58016067430, 58016067460, 58016067490, 58016073600, 58016073602, 58016073630, 58016073660, 58016073690, 58864046315, 58864046330, 58864064860, 58864064890, 58864071930, 58864071990, 58864087830, 59746038306, 59746038309, 59746038310, 59746038406, 59746038409, 59746038410, 59746038506, 59746038509, 59746038510, 59746038606, 59746038609, 59746038610, 60346033030, 60346037030, 60346043630, 60346043830, 60429070730, 60429070790, 60429070830, 60429070890, 60429070930, 60429070990, 60429071030, 60429071090, 60505011500, 60505011505, 60505011600, 60505011605, 60505011607, 60505011700, 60505011705, 60505011707, 60505011800, 60505011805, 63304066201, 63304066205, 63304066301, 63304066305, 63304066401, 63304066405, 63304066501, 63304066505, 63629141401, 63629141402, 63739028715, 63739028815, 63739028915, 63739042410, 63739042510, 63739042610, 68115064700, 68258603503, 68258604503, 68258604509, 68258606509, 68258610109

Tamsulosin
00093733801, 00115821101, 00115821103, 00173080913, 00173080959, 00228299611, 00228299650, 00378250010, 00378250077, 00378250093, 00591377119, 00591377130, 00597005801, 00597016601, 00781207601, 00904615661, 00904640161, 00904640180, 00904640189, 10370016901, 10370016905, 10370028009, 10370028011, 10544028030, 16590029810, 16590029820, 16590029830, 16590029840, 16590048904, 16590048930, 16590048960, 16590048971, 16590048990, 16714071301, 16714071302, 33261083907, 33261083930, 33261083990, 35356079330, 35356079390, 42254001203, 42254001210, 42254001230, 42254001290, 43063026401, 43063026430, 43063026490, 43063072530, 49999088100, 49999088130, 51079029401, 51079029417, 51079029419, 51079029420, 51079029430, 51079029456, 51655067726, 52959094710, 53217015407, 53217015430, 53217015490, 53217015907, 53217015930, 53217015990, 54569476800, 54569476801, 54569476802, 54569617400, 54569617401, 54868435600, 54868435601, 54868435602, 54868435603, 54868435604, 54868443700, 54868443701, 54868443702, 54868443703, 55048078530, 55700017630, 55700017690, 55700018630, 55700018690, 55700020030, 55700020090, 55887030530, 55887030560, 57237001401, 57237001405, 58016041000, 58016041030, 58016041060, 58016041090, 58864088230, 60429010601, 60429010605, 60429010690, 60760098230, 60760098290, 62756016013, 62756016081, 62756016088, 63187035830, 63187035890, 63187037107, 63739056710, 63739081141, 63739081143, 63739081145, 64679051602, 64679051603, 65862059801, 65862059805, 66105099203, 66116014530, 66267069330, 66267069360, 66336090207, 66336090210, 66336090230, 66336090290, 66993058030, 66993058085, 68071084930, 68084029901, 68084029911, 68084040701, 68115054200, 68115054290, 68115071290, 68382013201, 68382013210

Levodopa or carbidopa
00004005401, 00004005414, 00004005501, 00004005514, 00004005601, 00004005614, 00004005701, 00004005714, 00004007201, 00004010301, 00006391568, 00006391668, 00006391768, 00006391868, 00006391968, 00056025168, 00056027168, 00056027268, 00056051168, 00056052128, 00056052168, 00056052185, 00056060128, 00056060168, 00056064728, 00056064768, 00056065028, 00056065068, 00056065428, 00056065468, 00074301207, 00078040705, 00078040805, 00078040905, 00078052705, 00078054405, 00078054505, 00091334101, 00091334201, 00091334301, 00093029201, 00093029205, 00093029301, 00093029305, 00093029310, 00093029401, 00093029405, 00093029410, 00115391101, 00115391102, 00115392201, 00115392202, 00182194801, 00182194805, 00182194889, 00182194901, 00182194905, 00182194910, 00182194989, 00182195001, 00182195005, 00182195010, 00182195089, 00228253810, 00228253850, 00228253910, 00228253950, 00228253996, 00228254010, 00228254050, 00228254096, 00364253801, 00364253901, 00364254001, 00378007801, 00378008501, 00378008801, 00378009401, 00378113301, 00378505101, 00378505201, 00378505301, 00378830001, 00378830101, 00378830201, 00378830301, 00378830401, 00378830501, 00403419101, 00405413201, 00405413301, 00405413401, 00536555501, 00536555601, 00536555701, 00580020101, 00603256821, 00603256921, 00603257021, 00615353713, 00615353729, 00615353743, 00615353753, 00615353763, 00615353765, 00615356113, 00615356129, 00615356143, 00615356153, 00615356163, 00615356165, 00615450429, 00615450443, 00615450453, 00615450463, 00615450465, 00615456853, 00615456863, 00677149301, 00677149401, 00677149501, 00781162601, 00781162613, 00781162701, 00781162713, 00781162801,

00781162813, 00781561301, 00781562501, 00781563701, 00781564101, 00781565401, 00781566901, 00839776506, 00839776606, 00839776706, 00904574961, 00904575061, 00904623761, 00904623861, 00904771860, 00904771861, 00904771960, 00904771961, 00904772060, 00904772061, 12280011700, 16729007801, 16729007817, 16729007901, 16729007917, 18860034101, 18860034201, 18860034301, 21695050530, 21695050560, 21695061130, 25010071115, 33261071930, 33261071990, 33261072030, 33261072090, 35356035530, 35356035560, 35356035590, 35356062860, 35356062960, 38779020203, 38779020204, 38779020205, 38779020208, 38779020210, 38779020215, 38779020225, 38779023503, 38779023504, 38779023505, 38779023508, 38779023510, 38779023515, 38779023525, 38779023550, 42291018801, 42799012301, 43975022010, 47335000188, 47335000288, 47335000388, 47335000488, 47335000588, 47335000688, 47335018688, 47335018788, 47335018888, 47781033201, 49452169201, 49452169202, 49452169203, 49452169204, 49452169205, 49452268001, 49452268002, 49452268003, 49452268004, 49452268005, 49999099860, 49999099960, 50436675302, 50962070057, 51079075501, 51079075520, 51079075601, 51079075617, 51079075619, 51079075620, 51079075624, 51079075657, 51079078301, 51079078320, 51079086101, 51079086120, 51079088401, 51079088417, 51079088419, 51079088420, 51079088501, 51079088520, 51079092301, 51079092317, 51079092319, 51079092320, 51079092357, 51079097801, 51079097820, 51079097857, 51552110202, 51552110204, 51552134803, 51552134804, 51862007701, 51862007705, 51862007901, 51862007905, 51862007910, 51862008001, 51862008005, 51862008010, 51927213900, 51927248000, 54092006001, 54092006101, 54092006201, 54569040500, 54569282200, 54569498800, 54569531700, 54569531701, 54569649100, 54868127001, 54868133400, 54868133401, 54868133402, 54868133404, 54868133405, 54868133406, 54868133407, 54868154401, 54868188200, 54868188201, 54868188202, 54868283400, 54868283401, 54868283402, 54868286600, 54868286601, 54868286602, 54868565900, 54868565901, 54868577602, 55048005230, 55048005330, 55048005430, 57480047601, 57480047606, 57480080701, 57480080706, 57480080801, 57480080806, 57866673501, 57866673601, 57866673701, 57866673801, 57866673901, 58016095800, 58016095820, 58016095830, 58016095840, 58016095856, 58016095890, 58177038304, 59075058510, 59075058710, 59591024668, 59591024768, 59591025068, 60429015201, 60429015301, 60429015310, 60429015401, 60429015405, 60429065001, 60429065005, 60429065101, 60429065105, 60429065201, 60429065205, 60505012801, 60505012802, 60505012901, 60505012902, 60505013001, 60505013002, 60505013100, 60505013101, 60505013200, 60505013201, 60809011355, 60809011372, 60809012555, 60809012572, 60951060368, 60951060568, 60951060585, 60951060768, 60951060785, 61392017730, 61392017731, 61392017732, 61392017739, 61392017745, 61392017751, 61392017754, 61392017756, 61392017760, 61392017790, 61392017791, 61392018030, 61392018031, 61392018032, 61392018039, 61392018045, 61392018051, 61392018054, 61392018056, 61392018060, 61392018090, 61392018091, 61392018330, 61392018331, 61392018332, 61392018339, 61392018345, 61392018351, 61392018354, 61392018356, 61392018360, 61392018390, 61392018391, 62584064101, 62584064201, 62584064233, 62584064301, 62756018688, 62756018788, 62756018888, 62756045788, 62756046188, 62756051713, 62756051788, 62756051813, 62756051818, 62756051888, 62756051913, 62756051988, 62991201801, 62991201802, 62991201803, 62991203901, 62991203902, 62991203903, 62991203904, 62991284801, 62991284802, 62991284803, 62991285601, 62991285602, 62991285603, 62991285604, 63370003915, 63370003925, 63370003935, 63629163801, 63739001310, 63739001710, 63739004601, 63739004602, 63739004603, 63739004610, 63739004615, 63739004701, 63739004702, 63739004703, 63739004704, 63739004710, 63739004715, 63739004801, 63739004803, 63739004810, 63739004815, 64679078202, 64679078204, 64679078302, 64679078304, 64679078402, 64679078404, 64679078502, 64679078504, 64679078602, 64679078604, 64679078702, 64679078704, 64896066101, 64896066143, 64896066201, 64896066243, 64896066301, 64896066343, 64896066401, 64896066443, 68001017100, 68001017200, 68084009201, 68084009301, 68084009311, 68084009401, 68084009411, 68084028101, 68084028111, 68084028201, 68084028211, 68115045460, 68258714003, 68258714109, 68682020025

© 2021 Simmering JE et al. *JAMA Neurology*.

eTable 1. Cohort sample size by exclusion step

	Denmark		Truven	
Step	Number of TZ/DZ/AZ	Number of Tamsulosin	Number of TZ/DZ/AZ	Number of Tamsulosin
All newly started on med	144,078	160,320	265,924	1,266,762
At least 2 dispensing events within 1st year of use	89,503	100,496	185,801	1,070,358
Age at least 40	87,704	99,211	180,693	598,353
At least 1 year from RX start until censoring or event, free of event at RX start	69,998	72,100	102,222	580,320
Matched	52,365	52,365	94,883	94,883

© 2021 Simmering JE et al. *JAMA Neurology*.

eTable 2. Demographic and baseline characteristics of the Danish cohort before and after matching

	Unmatched			Matched		
	**TZ/DZ/AZ**	**Tamsulosin**	**SMD**	**TZ/DZ/AZ**	**Tamsulosin**	**SMD**
N	68,998	72,100		52,365	52,365	
Age						
Median (IQR)	68 (61-75)	69 (62-76)	0.06	68 (61-75)	68 (61-75)	0.00
40-59 years	14,949 (21.7%)	13,739 (19.1%)	0.06	10,661 (20.4%)	10,542 (20.1%)	0.01
60-79 years	44,830 (65.0%)	47,970 (66.5%)	0.03	34,912 (66.7%)	34,938 (66.7%)	0.00
80+ years	9,219 (13.4%)	10,391 (14.4%)	0.03	6,792 (13.0%)	6,885 (13.1%)	0.01
Use of drugs (current use)						
Low-dose aspirin	15,148 (22.0%)	16,916 (23.5%)	0.04	12,372 (23.6%)	11,907 (22.7%)	0.02
Non-aspirin NSAIDs	10,296 (14.9%)	10,220 (14.2%)	0.02	7,764 (14.8%)	7,492 (14.3%)	0.01
5a-reductase inhibitors	3,716 (5.4%)	5,206 (7.2%)	0.08	2,736 (5.2%)	2,865 (5.5%)	0.01
Statins	13,140 (19.0%)	16,845 (23.4%)	0.11	11,709 (22.4%)	11,289 (21.6%)	0.02
SSRIs	3,541 (5.1%)	4,101 (5.7%)	0.02	2,930 (5.6%)	2,721 (5.2%)	0.02
Diagnoses						
Diabetes	8,576 (12.4%)	8,496 (11.8%)	0.02	6,509 (12.4%)	6,071 (11.6%)	0.03
COPD	4,106 (6.0%)	5,001 (6.9%)	0.04	3,300 (6.3%)	3,155 (6.0%)	0.01
HF	2,404 (3.5%)	2,545 (3.5%)	0.02	1,910 (3.6%)	1,788 (3.4%)	0.01
Alcohol-related disease	1,891 (2.7%)	1,992 (2.8%)	0.00	1,479 (2.8%)	1,395 (2.7%)	0.01
Charlson Comobidity Index						
0	46,254 (67.0%)	47,548 (65.9%)	0.02	34,386 (65.7%)	35,051 (66.9%)	0.03
1-2	17,131 (24.8%)	18,831 (26.1%)	0.03	13,604 (26.0%)	13,281 (25.4%)	0.01
3	5,613 (8.1%)	5,721 (7.9%)	0.01	4,375 (8.4%)	4,033 (7.7%)	0.02
Education						
<=10 years	22,051 (32.0%)	23,375 (32.4%)	0.01	16,730 (31.9%)	16,563 (31.6%)	0.01
11-12 years	26,281 (38.1%)	28,497 (39.5%)	0.03	20,507 (39.2%)	20,547 (39.2%)	0.00
13+ years	13,378 (19.4%)	14,710 (20.4%)	0.03	10,684 (20.4%)	10,741 (20.5%)	0.00
Unknown	7,288 (10.6%)	5,518 (7.7%)	0.10	4,444 (8.5%)	4,514 (8.6%)	0.00
Other markers						
No. of antihypertensives						

© 2021 Simmering JE et al. *JAMA Neurology*.

0	36,572 (53.0%)	38,858 (53.9%)	0.02	26,294 (50.2%)	29,233 (55.8%)	0.11
1-2	23,562 (34.1%)	26,134 (36.2%)	0.04	18,309 (35.0%)	18,319 (35.0%)	0.00
3-4	8,864 (12.8%)	7,108 (9.9%)	0.09	7,762 (14.8%)	4,813 (9.2%)	0.17

© 2021 Simmering JE et al. *JAMA Neurology*.

eTable 3. Demographic and baseline characteristics of the Truven cohort before and after matching

	Unmatched			Matched		
	**TZ/DZ/AZ**	**Tamsulosin**	**SMD**	**TZ/DZ/AZ**	**Tamsulosin**	**SMD**
N	102,222	374,014		94,883	94,883	
Age at RX Start (Years)	64 (11.13)	65 (11.45)	-0.08	63.87 (11.06)	63.89 (11.05)	0.01
Elixhauser CM (%)						
Alcohol	1,911 (1.87%)	8,473 (2.27%)	-0.02	1,790 (1.89%)	1,848 (1.95%)	0.00
Anemia	13,044 (12.76%)	57,429 (15.35%)	-0.05	12,034 (12.68%)	12,193 (12.85%)	0.00
Blood Loss	1,205 (1.18%)	6,153 (1.65%)	-0.03	1,115 (1.18%)	1,122 (1.18%)	0.00
CHF	7,988 (7.81%)	37,666 (10.07%)	-0.06	7,351 (7.75%)	7,447 (7.85%)	0.00
Coagulopathy	2,829 (2.77%)	15,228 (4.07%)	-0.05	2,574 (2.71%)	2,620 (2.76%)	0.00
Depression	6,625 (6.48%)	32,365 (8.65%)	-0.06	6,283 (6.62%)	6,431 (6.78%)	0.00
DM	28,607 (27.99%)	103,548 (27.69%)	0.00	26,467 (27.89%)	26,816 (28.26%)	-0.01
DM with Complications	10,685 (10.45%)	37,553 (10.04%)	0.01	9,691 (10.21%)	9,974 (10.51%)	-0.01
Drug Abuse	910 (0.89%)	4,596 (1.23%)	-0.02	856 (0.90%)	880 (0.93%)	0.00
Fluid or Electrolyte Disorder	9,908 (9.69%)	44,255 (11.83%)	-0.05	9,079 (9.57%)	9,364 (9.87%)	-0.01
HIV	337 (0.33%)	1,073 (0.29%)	0.01	315 (0.33%)	328 (0.35%)	0.00
HTN	69,004 (67.5%)	233,480 (62.43%)	0.08	62,726 (66.11%)	63,993 (67.44%)	-0.02
HTN with Complications	15,924 (15.58%)	50,744 (13.57%)	0.04	14,333 (15.11%)	14,749 (15.54%)	-0.01
Hypothyroidism	7,676 (7.51%)	36,007 (9.63%)	-0.05	7,270 (7.66%)	7,344 (7.74%)	0.00
Liver Failure	3,473 (3.40%)	17,502 (4.68%)	-0.05	3,308 (3.49%)	3,402 (3.59%)	0.00
Lymphoma	1,084 (1.06%)	5,243 (1.40%)	-0.02	1,021 (1.08%)	1,053 (1.11%)	0.00
Metastatic Cancer	1,115 (1.09%)	7,176 (1.92%)	-0.05	1,039 (1.10%)	1,008 (1.11%)	0.00
Other Neuro Disorders	5,652 (5.53%)	33,423 (8.94%)	-0.09	5,240 (5.52%)	5,327 (5.61%)	0.00
Obesity	7,446 (7.28%)	32,361 (8.65%)	-0.04	7,142 (7.53%)	7,390 (7.79%)	-0.01
Paralysis	1,495 (1.46%)	8,414 (2.25%)	-0.04	1,354 (1.43%)	1,394 (1.47%)	0.00
Pulmonary Circ. Disease	1,853 (1.81%)	11,038 (2.95%)	-0.05	1,694 (1.79%)	1,769 (1.86%)	0.00
Psychoses	5,247 (5.13%)	25,591 (6.84%)	-0.05	4,960 (5.23%)	4,971 (5.24%)	0.00
Peptic Ulcer Disease	237 (0.23%)	1,283 (0.34%)	-0.01	222 (0.23%)	213 (0.22%)	0.00

© 2021 Simmering JE et al. *JAMA Neurology*.

Chronic Pulmonary Disease	19,833 (19.40%)	92,021 (24.60%)	-0.09	18,853 (19.87%)	18,721 (19.73%)	000
Peripheral vascular disease	12,592 (12.32%)	59,378 (15.88%)	-0.07	11,774 (12.41%)	11,867 (12.51%)	0.00
Renal Failure	8,631 (8.44%)	25,094 (6.71%)	0.05	7,471 (7.87%)	7,924 (8.35%)	-0.01
RA	3,884 (3.80%)	18,850 (5.04%)	-0.04	3,681 (3.88%)	3,698 (3.90%)	0.00
Solid Tumor	11,608 (11.36%)	62,150 (16.62%)	-0.11	11,095 (11.61%)	10,846 (11.43%)	0.01
Valvular Disease	11,693 (11.44%)	57,109 (15.27%)	-0.08	11,015 (11.61%)	11,148 (11.75%)	0.00
Weight Loss	2,521 (2.47%)	15,705 (4.20%)	-0.07	2,327 (2.45%)	2,391 (2.52%)	0.00
RX Start Year	2008.49 (3.71)	2010.13 (3.95)	-0.30	2008.73 (3.64)	2008.88 (3.60)	-0.03
Mean Inpatient Days Per Year	0.97 (5.45)	1.70 (7.21)	-0.08	0.89 (4.50)	0.99 (4.35)	-0.02
Mean Inpatient Events Per Year	0.18 (0.54)	0.28 (0.66)	-0.11	0.18 (0.51)	0.18 (0.48)	-0.01
Outpatient Visits Per Year	15.14 (16.04)	17.24 (17.34)	-0.09	15.07 (15.82)	15.22 (14.76)	-0.01
Mean Years After Medication Start	3.76 (2.67)	3.71 (2.68)	0.01	3.78 (2.65)	3.78 (2.65)	0.00

© 2021 Simmering JE et al. *JAMA Neurology*.

eTable 4. Typical doses and duration of therapy by drug

Values are median and interquartile ranges. Note that the total number of enrollees taking each of the AZ, DZ, TZ is greater than the number of people taking AZ/DZ/TZ because of switching between AZ, DZ, and TZ within the same enrollee.

	Danish Cohort				Truven Cohort			
	**TZ**	**DZ**	**AZ**	**Tamsulosin**	**TZ**	**DZ**	**AZ**	**Tamsulosin**
Number	2,879	15,501	33,981	52,365	37,144	38,747	24,179	94,883
Total Defined Daily Doses	475 (130, 1,481)	1,044 (328, 2,426)	706 (237, 1,920)	780 (230, 1,920)	252 (72, 720)	345 (105, 870)	520 (160, 1,200)	450 (150, 1,020)

© 2021 Simmering JE et al. *JAMA Neurology*.

eFigure 1. Kaplan-Meier survival curves stratified by age at medication start

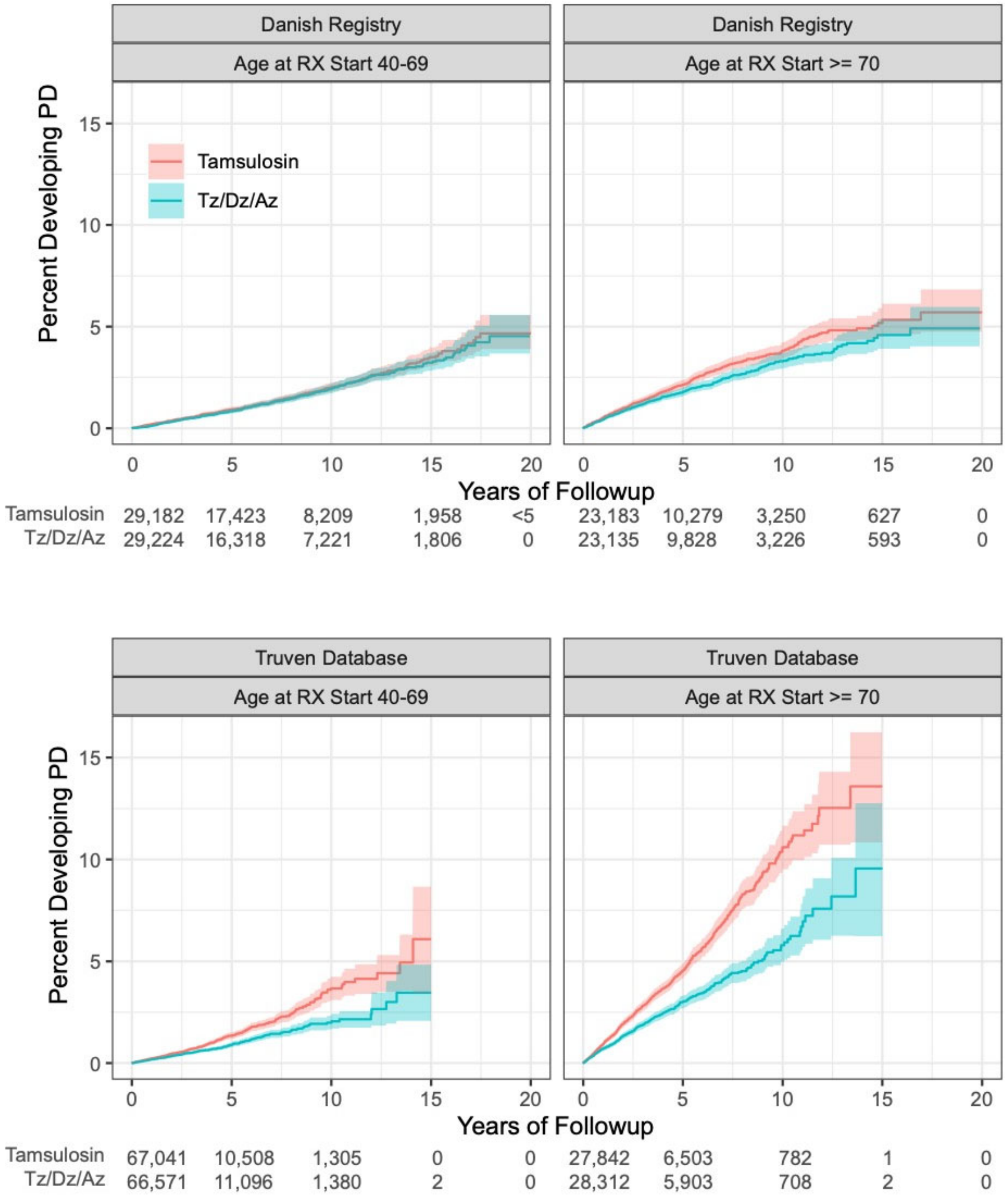

© 2021 Simmering JE et al. *JAMA Neurology*.

eFigure 2. Log(−log[survival]) plots

If the hazards are proportional, these curves should not cross and should be parallel. Crossing and deviations from parallel lines early is less of an issue than late crossings or deviations. These lines appear relatively parallel and only cross during the very early data phase when the noise is larger than the signal. This suggests the proportional hazards assumption is reasonable.

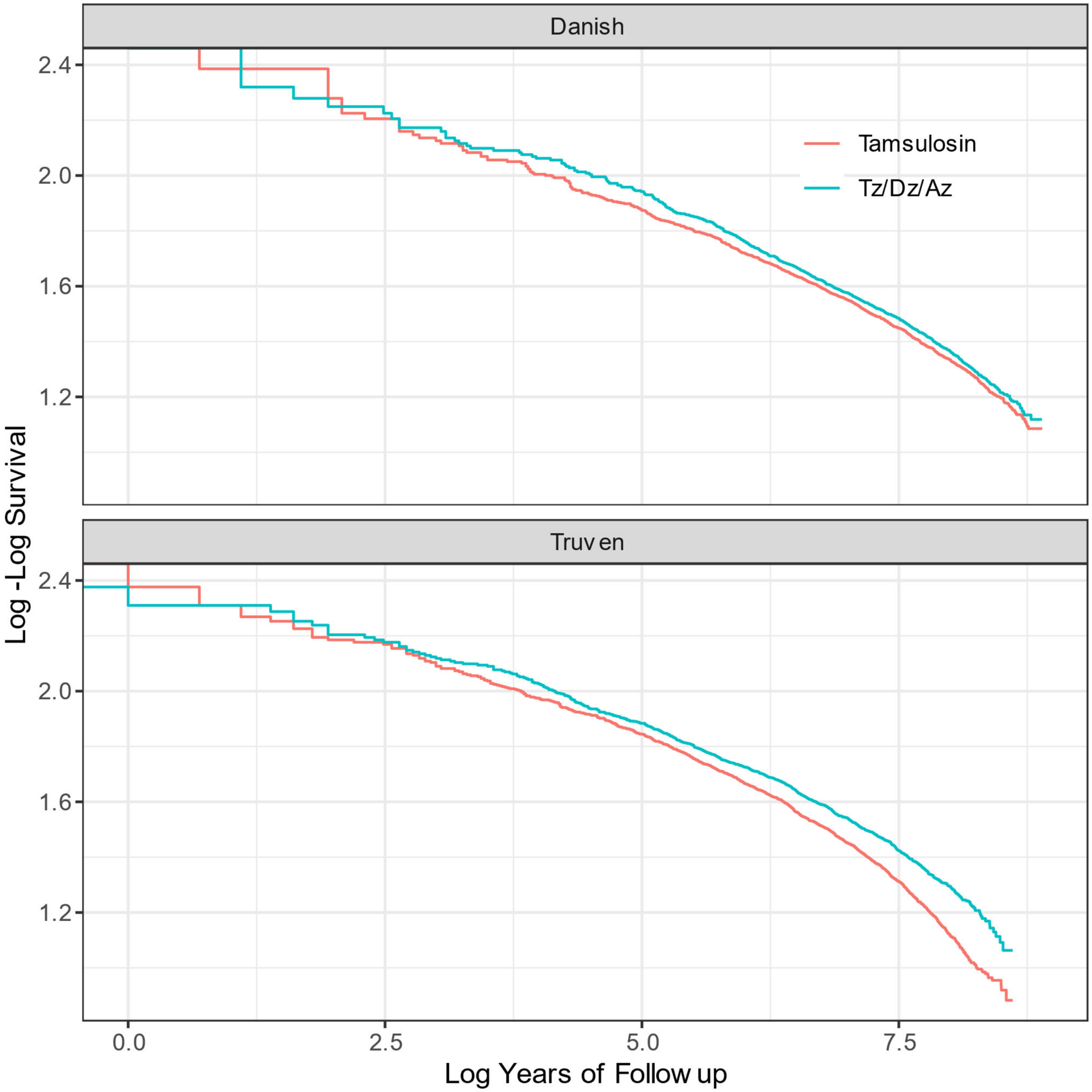

© 2021 Simmering JE et al. *JAMA Neurology*.
